# Reduced microbial diversity induces larger volatile organic compound emissions from soils

**DOI:** 10.1038/s41598-020-63091-8

**Published:** 2020-04-08

**Authors:** Letizia Abis, Benjamin Loubet, Raluca Ciuraru, Florence Lafouge, Sabine Houot, Virginie Nowak, Julie Tripied, Samuel Dequiedt, Pierre Alain Maron, Sophie Sadet-Bourgeteau

**Affiliations:** 1 Sorbonne Université, UPMC, Paris, France; 2 INRA, UMR ECOSYS, INRA, AgroParisTech, Université Paris-Saclay, 78850 Thiverval-Grignon, France; 3 INRA, UMR AgroEcologie, AgroSup Dijon, BP 87999, 21079 Dijon, cedex France; 4 Present Address: Technische Universität Berlin, Umweltchemie und Luftrinhaltunz, Straße des 17. Juni 135, Berlin, 10623 Germany

**Keywords:** Environmental chemistry, Environmental impact

## Abstract

Microorganisms in soil are known to be a source and a sink of volatile organic compounds (VOCs). The role of the microbial VOCs on soil ecosystem regulation has been increasingly demonstrated in the recent years. Nevertheless, little is known about the influence of the microbial soil community structure and diversity on VOC emissions. This novel study analyzed the effect of reduced microbial diversity in soil on VOC emissions. We found that reduced levels of microbial diversity in soil increased VOC emissions from soils, while the number of different VOCs emitted decreased. Furthermore, we found that *Proteobacteria*, *Bacteroidetes* and fungi phyla were positively correlated to VOC emissions, and other prokaryotic *phyla* were either negatively correlated or very slightly positively correlated to VOCs emissions. Our interpretation is that *Proteobacteria*, *Bacteroidetes* and fungi were VOC producers while the other prokaryotic *phyla* were consumers. Finally, we discussed the possible role of VOCs as mediators of microbial interactions in soil.

## Introduction

Recent studies have been focused on biogenic volatile organic compounds (bVOCs) released by aboveground biomass, because of their contribution to atmospheric chemistry^1–3^. Often, VOC fluxes by soil, and in particular by microorganisms, are not directly considered. A recent study showed that VOCs from vegetation could be overestimated compared to soil emissions by reporting comparable fluxes of methanol from bare soil and canopy in a maize crop^4^.

VOC emissions by soils may be boosted by organic amendment, due to soil organic matter (SOM) content and soil microbial biomass increase, compared to soil receiving mineral fertilization^5^. Among the different organic amendments, organic waste products (OWPs) resulting from human activities (*i.e*., sewage sludge, municipal solid waste composts, farmyard manure) are being increasingly used since they facilitate the recycling of nutrients and improve soil fertility. OWPs are also responsible for changing the microbial structure and increasing soil microbial diversity in soil^6–10^ due to the incorporation of exogenous microorganisms colonizing the organic amendment^11^. Microbial diversity in soil ensures several soil ecosystems functions such as (i) carbon (C) balance between organic C sequestration and C mineralization^12^, (ii) organic matter break down^13^, and more generally, (iii) nutrient recycling. Microorganisms are also directly involved in the production of VOCs emitted by soil^14^. Moreover, VOCs play a pivotal role in several processes such as the communication between organisms^15^, alterations of microbial nutrient cycles^16^ and growth rate of microorganisms colonizing the ecosystem^17^. Recently, it has been demonstrated that several microbial VOCs are involved in plant seed germination,plants growth^18^ and plant pathogens suppression^19^. The microbial community is not only a source of VOCs, but also a sink that microorganisms use as a source of energy^20^. Thanks to this characteristic, microorganisms are ideal to be used for biofiltration^21,22^. Biofiltration mechanisms consist in maintaining a mixed microbial community into biofilters in order to absorb and break down multiple VOCs^23^. The biofilters performance is particularly affected by the structure of the microbial community, the diversity and the interaction between microorganisms^24^, showing that VOC absorption depends the microbial community structure. VOC emissions can also depend on the taxa^25,26^ and thus, on the structure of the microbial community in soil. To our knowledge, no studies have reported the measurement of VOC emissions in response to soil microbial diversity loss and organic waste amendment in the soil. Hence, the effect of microbial diversity loss on VOC emissions is still unknown. Since microorganisms play a central role in soil VOC emissions, we hypothesized that the manipulation of the microbial community in soil might lead to some variations in the quantity and quality of VOC emissions.

The manipulation of the microbial diversity was performed by inoculating 30 g of sterile soils amended with 4 different organic waste products (OWPs): municipal solid waste compost derived from the composting of residual solid wastes after removing dry and clean packaging (MWS), farmyard manure (FYM), a compost derived from the co-composting of green wastes with sewage sludge (GWS) and the bio-waste compost derived from the co-composting of green wastes and source-separated organic fractions of municipal solid wastes (BIOW). All the samples were compared to a control (CN) with no OWPs. The inoculation for each soil was performed with three dilutions of a soil suspension taken from the same soil sampled before sterilization. After six weeks of incubation at 20 °C, VOC emissions were measured with a dynamic chamber method using a highly sensitive proton transfer, quadrupole injection, time of flight mass spectrometer (PTR-Qi-TOF-MS, Ionicon). The VOC concentrations were measured every second for 3 min for each sample (Samples = 45, replicates = 3). The mass spectra were analyzed using PTR-MS viewer-3 software in order to integrate each ion peak. The microbial diversity was measured after VOC emission measurements by a high throughput sequencing approach targeting 16S and 18S ribosomal genes.

## Results

### Soil microbial biomass

After six weeks of incubation, the microbial biomass was not significantly different among microbial dilution levels for each type of amended treatment except for FYM (soil amended with farmyard manure), where the highest dilution level had the highest microbial biomass (Table 1). A normalization of the VOC emissions by the microbial biomass levels showed no significant difference in the variation of VOC emissions with dilution compared to non-normalized data. We could attribute the variation of VOC emissions measured in the different dilution treatments to microbial diversity and not to microbial biomass quantity. Whatever the dilution levels considered, the microbial biomass was statistically higher in MWS treatment, FYM and GWS than in BIOW and CN.

**Table 1:** Effect of different microbial diversity dilution on soil prokaryotic and fungal diversity indices (Shannon and Richness) and density of the genes copies of fungi and prokatyiotes (18S and 16S genes copies).

Organic Waste Product	Level of diversity	Biomass µg DNA g sol^−1^ ± sd	Indices of diversity				Density		F/P ratio
			Shannon (Prokaryotes)	Richness (Prokaryotes)	Shannon (Fungi)	Richness (Fungi)	16S gene copies per g of dry soil × 10^9^ ± sd × 10^9^	18S gene copies per g of dry soil × 10^8^ ± sd × 10^8^	
Control without organic input	D0	13.52 ± 2.41 a	4.44 ± 0.45a	1308 ± 247.67a	3.30 ± 0.16a	553.00 ± 111.53a	3.59 ± 1.56a	2.34 ± 1.31a	0,065
	D1	12.48 ± 2.09 a	4.06 ± 0.41ab	1083.67 ± 189.74ab	3.16 ± 0.51a	506.67 ± 152.75a	3.54 ± 0.641a	1.45 ± 0.332ab	0,041
	D2	12.69 ± 2.30 a	3.47 ± 0.16b	876.67 ± 88.29b	2.95 ± 0.84a	708.67 ± 207.61a	4.24 ± 1.73a	0.346 ± 0.281b	0,008
Biowaste	D0	11.24 ± 1.07 a	4.16 ± 1.31a	1751 ± 450.85a	3.27 ± 0.31b	533.33 ± 77.02b	2.97 ± 0.548a	2.49 ± 0.695a	0,084
	D1	15.83 ± 1.33 a	4.03 ± 0.35a	1346 ± 224.31ab	2.93 ± 0.42b	467.33 ± 93.50b	5.96 ± 0.148a	1.67 ± 0.929ab	0,028
	D2	17.38 ± 2.77 a	3.5 ± 0.30a	931 ± 3.5b	4.62 ± 0.26a	1184.33 ± 106.33a	6.87 ± 2.40a	0.129 ± 0.0548b	0,002
Farmyard manure	D0	16.25 ± 4.35 a	4.50 ± 0.13a	1462.67 ± 49.56a	3.83 ± 0.22a	746.33 ± 82.31a	3.78 ± 1.30b	1.93 ± 0.172b	0,051
	D1	21.22 ± 2.20 ab	4.11 ± 0.19a	1049.67 ± 97.03b	3.03 ± 0.14b	423.00 ± 40.01b	5.99 ± 1.67b	3.75 ± 0.294a	0,063
	D2	28.52 ± 3.48 b	3.24 ± 0.15b	718.67 ± 31.5c	4.58 ± 0.25a	1018.67 ± 173.37a	13.90 ± 2.93a	0.187 ± 0.0476c	0,001
Green waste and sludge	D0	18.30 ± 1.18 a	4.35 ± 0.10a	1442 ± 90.7a	2.93 ± 0.20ab	508.67 ± 28.75ab	5.02 ± 1.19b	6.51 ± 1.94a	0,129
	D1	20.26 ± 4.78 a	4.08 ± 0.12a	1153.67 ± 91.36ab	2.85 ± 0.22b	400.00 ± 53.07b	5.60 ± 0.746b	2.69 ± 0.812b	0,048
	D2	21.99 ± 3.77 a	3.42 ± 0.05b	961.33 ± 156.67b	4.67 ± 0.66a	1123.67 ± 325.92a	10.50 ± 2.23a	0.140 ± 0.0186c	0,001
Municipal solid waste	D0	22.31 ± 2.12 a	4.56 ± 0.13a	1579.00 ± 131.19a	2.86 ± 0.34a	514.67 ± 112.21a	6.70 ± 0.895b	7.92 ± 2.44a	0,118
	D1	21.52 ± 1.19 a	4.00 ± 0.13b	1114.00 ± 67.02b	2.68 ± 0.37a	380.67 ± 96.84a	6.93 ± 0.735b	3.70 ± 1.57a	0,053
	D2	23.97 ± 1.39 a	3.39 ± 0.24c	798.33 ± 39.80c	3.02 ± 1.90a	729.33 ± 379.96a	11.00 ± 2.06a	2.20 ± 3.44a	0,02

Letters indicate significant differences according to the Tukey test with p.value > 0.05. F/P ratio represent the fungi-to-prokaryotic ratio.

### Microbial diversity

**Figure 1:**
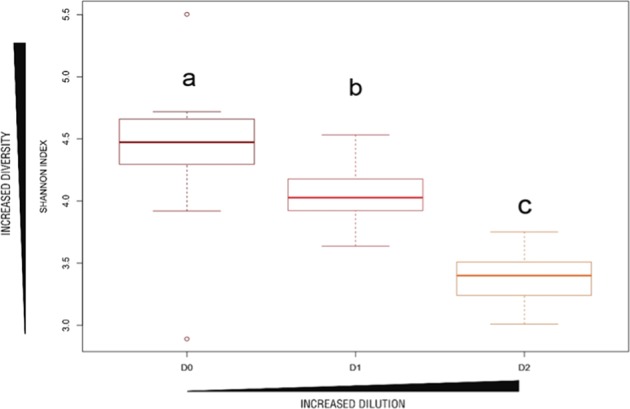
Shannon index for prokaryotes in the soil as a function of dilution levels. D0: no dilution of the microbial diversity. D1: microbial diversity dilution equal to 10^−3^. D2: microbial diversity dilution equal to 10^−5^. Bold line = median, boxes = interquartile, whiskers = minimum and maximum. Point = Shannon Index of each sample. Letters indicate significant differences according to the Tukey test with p-value < 0.05.

The Shannon index is a measure of microbial diversity and it has been calculated for both fungi and prokaryotic community in soil. The prokaryotic Shannon index shows a significant decrease of prokaryotic diversity with increasing dilution (p.value < 0.05) in our samples (Fig. 1). These results confirm the efficiency of the prokaryotic diversity manipulation. The dilution of the prokaryotic diversity was also efficient for the FYM samples, even though a larger biomass was found for the highest dilution (D2, Table 1). For fungi, the Shannon index revealed a decrease of fungal diversity in the higher dilution level (Fig. 1). In our microcosms, the fungi-to-prokaryotes (F/P) ratio was smaller (between 0.05 and 0.20) compared to the F/P ratio previously observed in similar studies^27^ where it was always higher than 0.20. Even if the portion of fungi in our microcosms seems to be negligible compared to prokaryotes, if we consider the density of gene copies of 18S, which represent the density of fungi, we notice that their density is between 3.75 × 10^8^ and 1.4 × 10^7^ (Table 1). This density might be consistent in all soils and might influence the VOC emissions, and the reason why we did not ignore this part of the microcosms.

A decrease of F/P ratio throughout the diversity gradient was also observed (Table 1**)** suggesting that while the microbial dilution was higher, the fungal community was disadvantaged compared to prokaryotic community.

**Figure 2:**
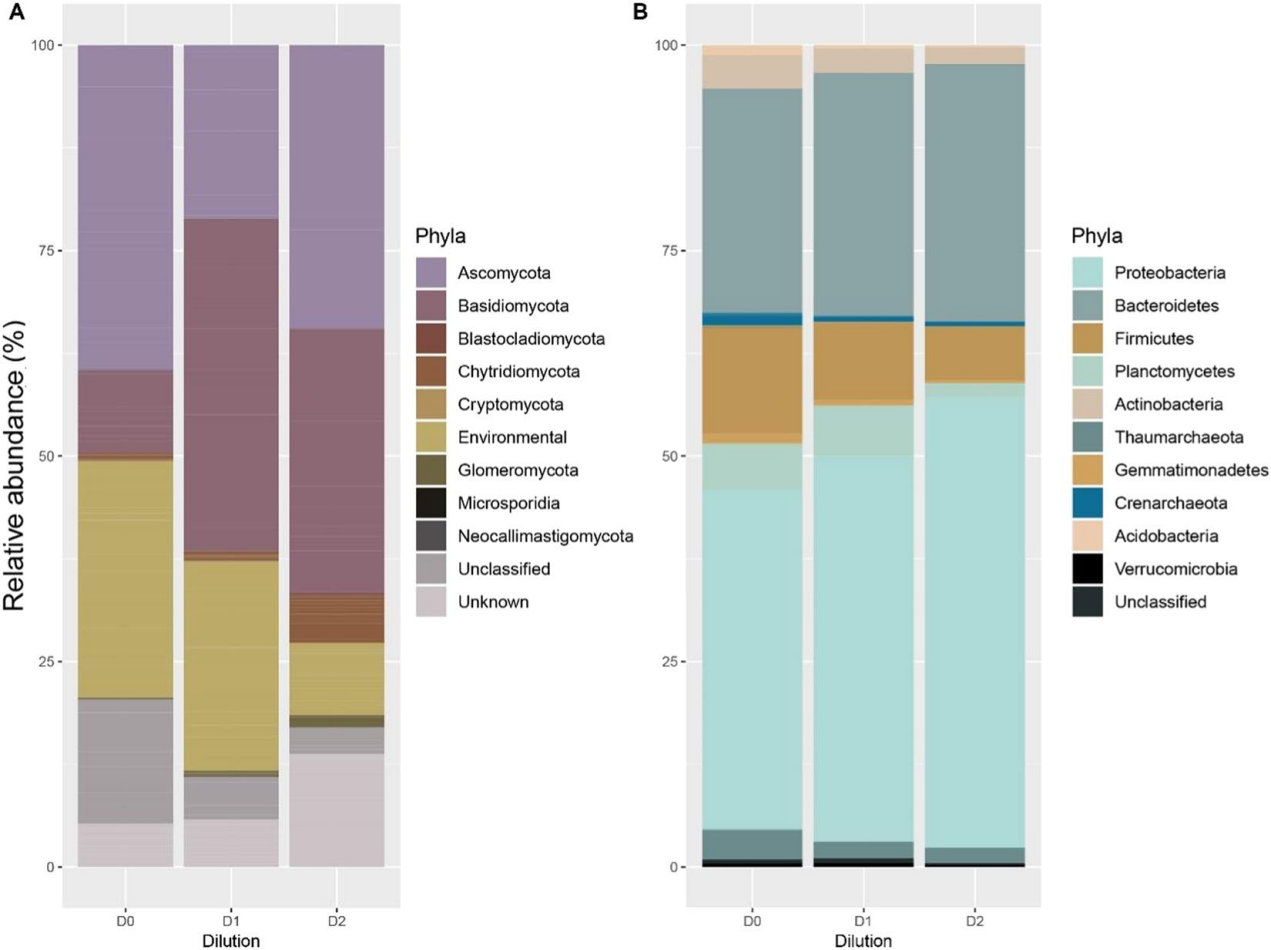
Relative abundances of the phyla. (**a**) Relative abundances of the prokaryotic phyla. (**b**) Relative abundances of the fungal phyla. D0: no dilution equal of the microbial diversity. D1: microbial diversity dilution equal to 10^−3^. D2: microbial diversity dilution equal to 10^−5^.

We observed the opposite pattern for fungi: *Basidiomycota*, *Chytridiomycota, Glomeromycota*, and *Neoclimastigomycota* were more abundant in the highest dilution levels (D2 samples) than in the lowest ones (D0 samples) although they were not the most abundant fungi in D0 (Fig. 2b). The opposite trend was observed for *Ascomycota* and *Blastoclamidiomycota*, which were the most abundant in D0.

As expected, the diversity manipulation tended to strengthen the presence of the most abundant prokaryotic phyla and reduce the presence of the less abundant ones. This was indeed observed with *Proteobacteria* and *Bacteroides* phyla which were the most abundant in the lowest dilution samples and showed an increased abundance in the highest dilution samples. *Firmicutes, Crenarchaeota, Actinobacteria, Thaumarchaeota, Planctomycetes, Gemmatimonadetes*, and *Acidobacteria* showed the opposite behavior (Fig. 2a**)**.

### VOC emissions from manipulated soils

**Figure 3:**
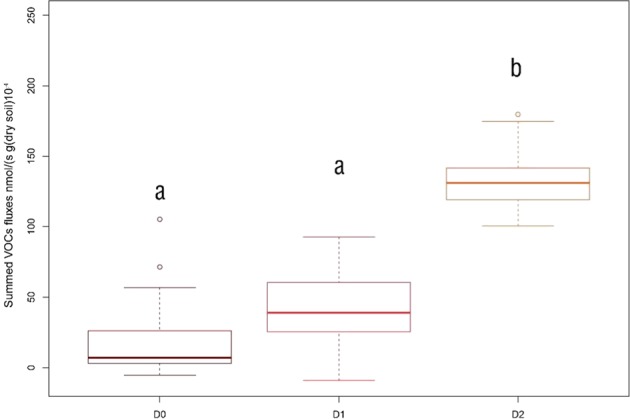
Summed VOCs fluxes rate as a function of microbial dilution in soils. Bold line = median, boxes = interquartile, whiskers = minimum and maximum, colored circles = outliers, grey dots = individual values. Letters indicate significant differences according to the Tukey test with p.value < 0.05. D0: no dilution equal of the microbial diversity. D1: microbial diversity dilution equal to 10^−3^. D2: microbial diversity dilution equal to 10^−5^.

We detected 754 ion peaks, spanning m/z 15 to m/z 510. VOC emissions summed among all detected ion peaks increased while the microbial diversity in the soil was lower (Fig. 3).

**Figure 4:**
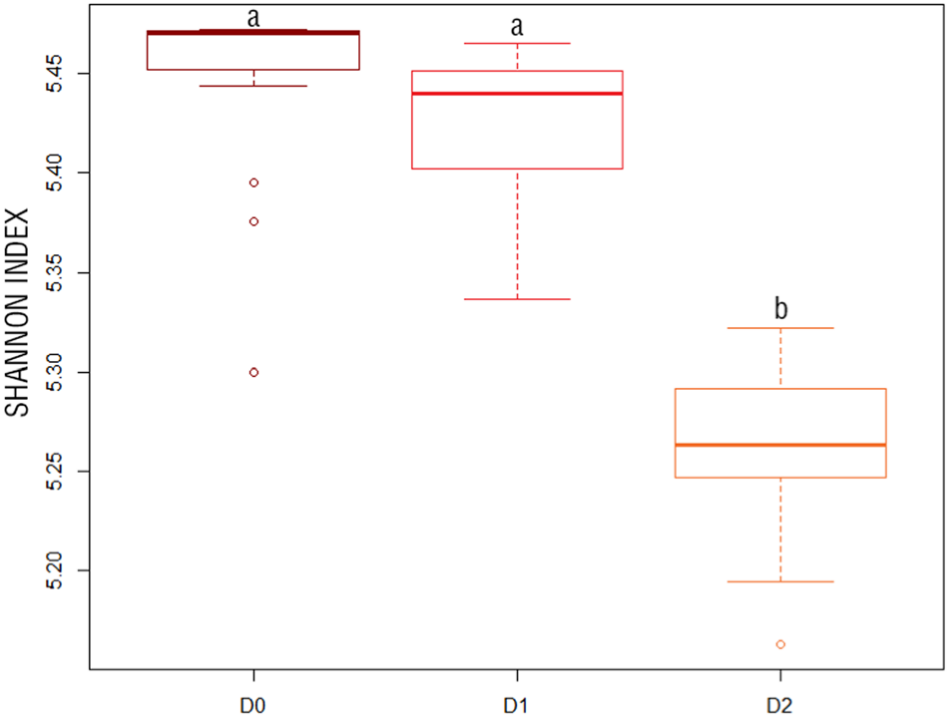
Shannon index for VOCs emissions in soil. D0 = microbial diversity pure or 10^0^, D1 = microbial dilution diversity equal to 10^−3^, D2 = microbial dilution diversity equal to 10^−5^. Letters indicate significant differences according to the Tukey test with p.value < 0.05.

The lower microbial diversity level showed a summed VOC flux between 0.5 and 3 times higher than the flux at the higher microbial diversity levels. This was true for all the considered soils. This means that, in our experiment, the effect of microbial dilution in driving soil VOC emissions overpassed the effect of OWPs amendment (Figs. S1 and S2, Tukey test on OWPs). The diversity in VOCs (Shannon index on VOCs emissions) was lowest in the lowest microbial diversity level, denoting that the larger summed emission rates were combined with a lower number of VOCs emitted (Fig. 4).

**Figure 5:**
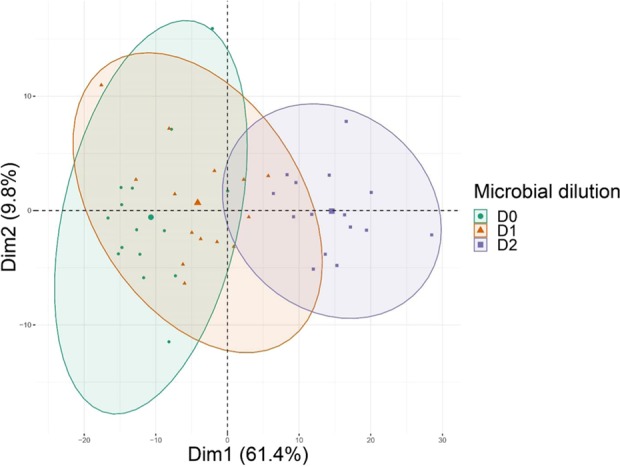
Effect of the different microbial dilutions on VOCs emissions by soil. The m/z of the 20 compounds that are explaining the variance in the two first components are shown on the graph. The percentage of the variance explained by the 2 first components is shown on each axis. The ellipses represent the similarity between samples. Samples in the same ellipsis are more similar than samples displayed in two different ellipses. D0: no dilution equal of the microbial diversity. D1: microbial diversity dilution equal to 10^−3^. D2: microbial diversity dilution equal to 10^−5^.

The diversity of the VOCs emitted (Table 1) also showed that the microbial dilution levels D0 and D1 had similar VOC profiles while the lowest microbial diversity level (D2) had a different profile (Fig. 5). Several VOC compounds explain the differences in VOC profiles between microbial dilutions. Tentative identification of the compounds explaining the variance in Fig. 5 are reported in Table 2, together with the 50 most emitted compounds in all dilution levels. These 65 compounds contribute almost 99% of the total emissions rate. The most emitted compounds were m/z 121.097 (tentatively identified as Propylbenzene, isopropylbenzene or 1,3,5-trimethylbenzene, Phenilacetaldheyde), m/z 135.113 (p-cymene) and m/z 73.062 (Butanone, MEK). Together, these compounds represented 70% of the summed VOCs emission rate (Table S2**)**. Acetaldehyde, butanone, and acetoin were emitted between 10 and 20 times more in samples with the highest dilution (D2) compared to samples with the lowest dilution (D0).

**Table 2:** Most emitted compounds for the different microbial dilution levels D0, D1 and D2 as well as compounds explaining most the variance in VOC emissions between dilution levels and those known as fungistatic VOCs.

m/z	Most likely formula	Tentative identification	Compounds class	Fungistatic compounds	Compounds most explaining the variance (PCA analysis)	Mean emission ± sd (nmol s^−1^ g^−1^(DW)) × 10^4^			Mean emission rates ± sd (nmol s^−1^ g^−1^(DW)) × 10^4^	Percentage contribution (% mol)		
						D0	D1	D2		D0	D1	D2
m121,097	C_9_H_12_	Propylbenzene cumene mesytilene 1,3,5-trimethylbenzene, Phenilacetaldheyde	Aromatic	Phenilac-etaldheyde^66^		7,83 ± 13,04	14,34 ± 11,04	49,59 ± 8,2	23,92 ± 17,11	52,37	49,41	54,95
m135,113	C_10_H_14_	p-cymene	Aromatic		x	2,04 ± 2,77	4,39 ± 2,44	10,45 ± 2,35	5,63 ± 3,22	13,65	15,13	11,58
m73.062	C_4_H_8_O	Butanone, MEK	Carbonyl- Ketone			0,82 ± 1,02	0,96 ± 1,49	2,49 ± 1,36	1,42 ± 0,71	5,49	3,32	2,75
m122.100	C_8_H_11_N	Benzenamine/2,6-Xylidine	Nitrogen compounds			0,75 ± 1,31	1,37 ± 1,12	5,23 ± 1,09	2,45 ± 1,85	5,04	4,72	5,79
m57.068	C_4_H_8_	Butene	Hydrocarbon - Alkene			0,51 ± 0,77	2 ± 0,81	3,11 ± 0,55	1,87 ± 0,91	3,43	6,89	3,44
m41.037	C_3_H_4_	Propyne	Hydrocarbon - Alkyine			0,35 ± 0,37	0,94 ± 0,55	2,19 ± 0,47	1,16 ± 0,69	2,32	3,25	2,43
m119.078	C_8_H_6_O		Oxygenated compound			0,31 ± 0,48	0,61 ± 0,42	1,88 ± 0,39	0,93 ± 0,63	2,07	2,09	2,08
m120.090	C_8_H_9_N		Nitrogen compounds			0,29 ± 0,53	0,52 ± 0,45	2,03 ± 0,53	0,95 ± 0,72	1,94	1,81	2,25
m105.066	C_5_H_12_S	Butane 1-methylthio, styrene	Hydrocarbon - Alkane			0,26 ± 0,43	0,47 ± 0,35	1,66 ± 0,34	0,8 ± 0,57	1,71	1,63	1,84
m136.021	C_7_H_5_NS	Benzothiazole	Aromatic heterocyclic compound	x^66^		0,23 ± 0,07	0,3 ± 0,15	0,4 ± 0,12	0,31 ± 0,06	1,52	1,05	0,44
m91.072	C_4_H_10_S	Diethyl sulphide	Organosulfur	x^66^		0,19 ± 0,31	0,33 ± 0,27	0,88 ± 0,25	0,47 ± 0,28	1,26	1,14	0,97
m136.118	C_10_H_15_		Hydrocarbon		x	0,16 ± 0,23	0,35 ± 0,2	0,87 ± 0,21	0,46 ± 0,27	1,08	1,20	0,97
m94.070						0,14 ± 0,21	0,27 ± 0,18	0,89 ± 0,17	0,43 ± 0,3	0,93	0,93	0,98
m79.071	C_6_H_6_	Benzene	Aromatic		x	0,12 ± 0,16	0,27 ± 0,18	0,9 ± 0,15	0,43 ± 0,31	0,83	0,93	1,00
m108.084	C_7_H_9_N	Dimethyl pyridine	Nitrogen compounds			0,1 ± 0,15	0,18 ± 0,12	0,66 ± 0,14	0,31 ± 0,23	0,66	0,61	0,73
m33.033	CH_4_O	Methanol	Alcohol			0,06 ± 0,04	0,08 ± 0,05	0,18 ± 0,12	0,1 ± 0,05	0,37	0,29	0,19
m134.108	C_9_H_11_N		Amine			0,05 ± 0,08	0,12 ± 0,07	0,28 ± 0,08	0,15 ± 0,08	0,37	0,41	0,31
m55.051	C_4_H_6_	1,3 butadiene	Hydrocarbon - Diene		x	0,05 ± 0,07	0,08 ± 0,13	0,27 ± 0,14	0,13 ± 0,09	0,35	0,27	0,30
m106.073	C_8_H_9_		Hydrocarbon			0,05 ± 0,08	0,08 ± 0,06	0,32 ± 0,07	0,15 ± 0,11	0,31	0,28	0,36
m133.108	C_10_H_13_/C_5_H_12_N_2_O_2_		Hydrocarbon/Amine			0,04 ± 0,05	0,09 ± 0,04	0,17 ± 0,03	0,1 ± 0,05	0,30	0,30	0,19
m122.064	C_7_H_8_NO		Amide			0,04 ± 0,06	0,08 ± 0,05	0,14 ± 0,02	0,08 ± 0,03	0,25	0,27	0,15
m95.052	C_6_H_6_O	Phenol	Oxygenated Aromatic		x	0,03 ± 0,04	0,06 ± 0,04	0,2 ± 0,03	0,1 ± 0,07	0,23	0,22	0,22
m74.065	C_3_H_7_NO	Dimethylformamide/Propanamide	Amide		x	0,03 ± 0,04	0,04 ± 0,06	0,1 ± 0,05	0,05 ± 0,03	0,21	0,13	0,11
m95.043	C_2_H_6_S_2_	Dimethyl disulfide	Organosulfur	x^66,67^		0,03 ± 0,04	0,06 ± 0,04	0,18 ± 0,03	0,09 ± 0,06	0,21	0,21	0,20
m123.073	C_8_H_11_O		Oxygenated compound			0,03 ± 0,04	0,05 ± 0,04	0,17 ± 0,02	0,08 ± 0,06	0,20	0,18	0,18
m45.033	C_2_H_4_O	Acetaldehyde	Carbonyl - Aldheyde			0,03 ± 0,29	0,06 ± 0,47	0,42 ± 0,45	0,17 ± 0,17	0,19	0,19	0,46
m122.062	C_8_H_9_O	Acetophenone	Aromatic ketone			0,03 ± 0,04	0,06 ± 0,03	0,1 ± 0,02	0,06 ± 0,02	0,17	0,20	0,11
m92.056	C_6_H_5_N		Heterocyclic compound			0,03 ± 0,04	0,05 ± 0,04	0,14 ± 0,03	0,07 ± 0,04	0,17	0,19	0,15
m58.071	C_4_H_9_		Hydrocarbon			0,02 ± 0,03	0,08 ± 0,03	0,12 ± 0,02	0,07 ± 0,03	0,14	0,27	0,13
m83.047	C_5_H_7_O		Oxygenated compound			0,02 ± 0,01	0,03 ± 0,02	0,17 ± 0,12	0,07 ± 0,07	0,12	0,10	0,19
m158.162	C_9_H_19_NO		Amide			0,02 ± 0,02	0,02 ± 0,02	0,02 ± 0,02	0,02 ± 0	0,12	0,07	0,02
m109.099	C_8_H_6_O	Benzofuran	Oxygenated Aromatic			0,01 ± 0,01	0,02 ± 0,01	0,05 ± 0,01	0,03 ± 0,01	0,10	0,07	0,05
m149.130	C_6_H_12_O_4_/C_10_H_12_O		Oxygenated compound			0,01 ± 0,01	0,03 ± 0,01	0,05 ± 0,01	0,03 ± 0,01	0,09	0,10	0,05
m95.083	C_5_H_6_N_2_	Methyl pyrazine	Hydrocarbon	x^66^		0,01 ± 0,01	0,02 ± 0,01	0,06 ± 0,01	0,03 ± 0,02	0,09	0,07	0,06
m111.041	C_7_H_10_O	2,3,5-trimethyl- Furan	Heterocyclic compound			0,01 ± 0,01	0,02 ± 0,01	0,02 ± 0,01	0,02 ± 0	0,09	0,06	0,02
m95.028	C_2_H_6_O_2_S		Organosulfur			0,01 ± 0,01	0,03 ± 0,02	0,1 ± 0,02	0,05 ± 0,03	0,09	0,10	0,11
m108.953						0,01 ± 0	0,01 ± 0	0,01 ± 0	0,01 ± 0	0,08	0,04	0,01
m117.084	C_6_H_12_O_2_	Ethyl Butyrate	Oxygenated compound			0,01 ± 0,01	0,02 ± 0,01	0,06 ± 0,01	0,03 ± 0,02	0,08	0,08	0,07
m61.049	C_2_H_4_O_2_	Acetic acid	Carboxylic acid			0,01 ± 0,01	0,01 ± 0,02	0,05 ± 0,05	0,02 ± 0,02	0,08	0,04	0,05
m80.048						0,01 ± 0,02	0,03 ± 0,02	0,09 ± 0,02	0,04 ± 0,03	0,08	0,09	0,09
m105.031	C_7_H_4_O		Heterocyclic compound			0,01 ± 0,02	0,02 ± 0,01	0,06 ± 0,01	0,03 ± 0,02	0,08	0,08	0,07
m53.037	C_4_H_4_		Hydrocarbons			0,01 ± 0,02	0,03 ± 0,02	0,1 ± 0,02	0,04 ± 0,03	0,08	0,09	0,11
m89.056	C_4_H_8_O_2_	Ethyl acetate/Butanoic acid/Acetoin	Acetate ester/Carboxylic acid			0,01 ± 0,02	0,08 ± 0,07	1,96 ± 1,68	0,68 ± 0,85	0,06	0,26	2,17
m118.089	C_5_H_11_NO_2_	Valine	Amino acid			0,01 ± 0,01	0,02 ± 0,01	0,05 ± 0,01	0,03 ± 0,02	0,06	0,06	0,06

**Figure 6:**
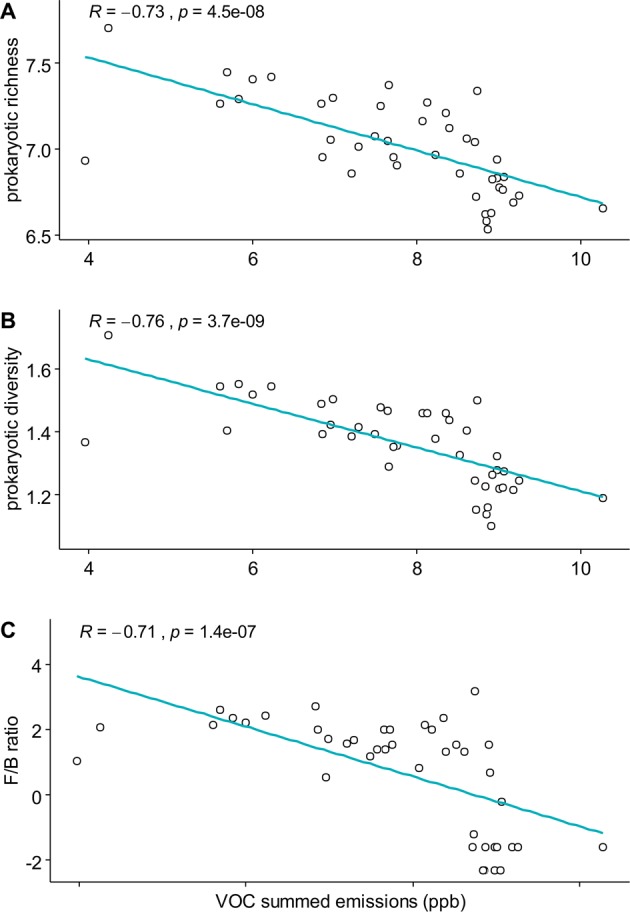
Correlation between log (VOC summed emissions) and (**a**) log (prokaryotic richness), (**b**) log (prokaryotic diversity), (**c**) log (F/P ratio). The Spearman correlation coefficient (R) and the p.value (*p*) are also displayed for all correlations.

The correlation between the summed VOC emissions from soil and the prokaryotic diversity, richness and the F/P ratio showed a significant negative correlation (Fig. 6) with a Spearman coefficient always lower than −0.71. Non-significant correlations were found for all other combinations (VOC diversity and fungal richness, fungal diversity and VOC summed emissions, and fungal richness and fungal diversity, Fig. S3. The variance partitioning showed that Prokaryotic and fungal richness explained 33.35% of the variability of summed VOC emission rates (adjusted *R*^2^; *P* < 0.01).

**Figure 7:**
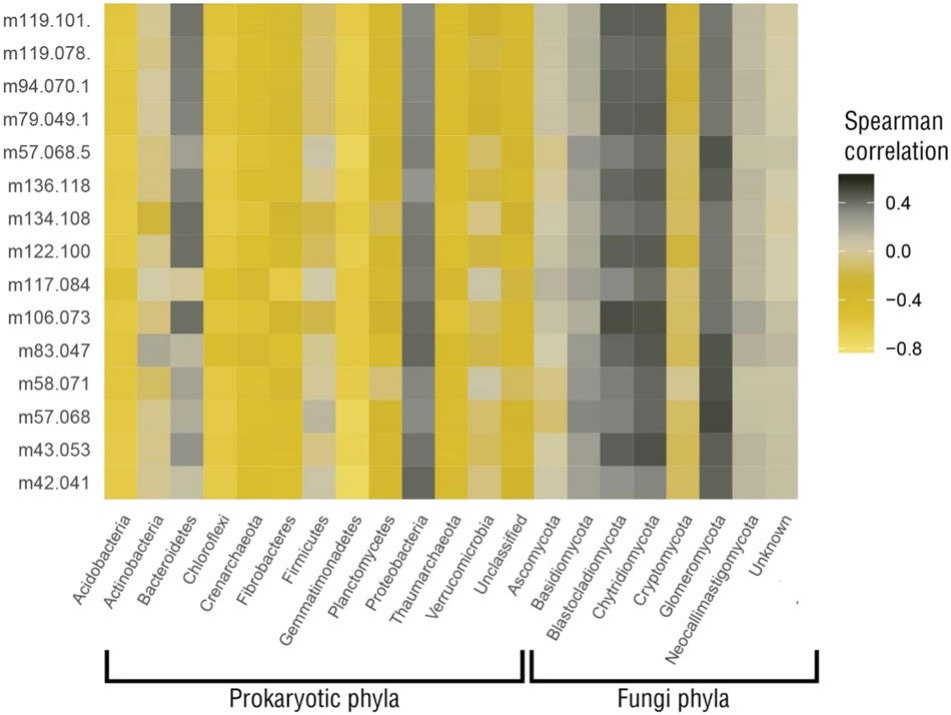
Correlation coefficients between VOCs emission rates and phyla abundance. Pearson correlation between log (emission rate + c) and log (phyla abundance + 1). The constant c was tuned to get always positive values. VOCs are named as ion mass to charge ratios (m/z). VOCs for which at least one correlation coefficient was larger than 0.6 with phyla are displayed.

Finally, we found that individual VOC emission rates were mostly negatively correlated with prokaryotic phyla abundance, except for *Bacteroidetes* and *Proteobacteria* whose abundances were positively correlated with almost all VOCs (Fig. 7). An opposite behavior was observed for the fungal phyla, where most had positive correlations with VOCs except for *Cryptomicota*.

## Discussion

### Microbial diversity

Tukey tests shows that the decrease of prokaryotic diversity index (Shannon index) was effective in all D2 samples compared to D0 (Fig. 1). This decrease can be explained as the result of a competition between slow-growing organisms such as *Acidobacteria*, *Actinobacteria*, *Planctomycetes*, and *Gemmatimonadetes*^28^, and fast-growing organisms, such as *Proteobacteria*, *Bacteroidetes* and *Firmicutes*^28^. Indeed, in reduced competition conditions^29^, fast-growing organisms (pioneer species) can colonize microcosms faster than others (slow-growing ones). The larger biomass observed in the highest dilution level (D2) in the FYM microcosms may be attributed to specific conditions of that soil treatment promoting even more the pioneering species.

### Microbial diversity affects VOCs total emission rates

Although significantly higher VOC emission rates from BIOW treatments have been reported in one of our previous studies^30^, in this study we noticed that the effect of the substrate on VOC emissions was negligible compared to the effect of the prokaryotic diversity (Figs. S1–S2). In our previous study^30^ we used the same initial soil samples without performing the microbial diversity manipulation and it was found that the most emitted compounds were acetone, butanone, and acetaldehyde. In this study we found that the masses m/z 121.097 (tentatively identified as propylbenzene, isopropylbenzene or 1,3,5-trimethylbenzene), m/z 135.113 (p-cymene) and m/z 73.062 (Butanone, MEK) were the most emitted ones. Acetone was not in the list of the 50 most emitted compounds which means that its contribution to the summed VOC emissions was lower than 0.01%. Acetaldehyde and butanone were emitted 100 and 10 times less, respectively, compared to our preview study^30^. The reason why the most emitted compound were this different it could be due to the different sample treatment. The samples used in our first study^30^ were not sterilized and thus the characteristics of the soils played a stronger role in the VOC emissions. For instance, acetone is one of the most emitted compound by agricultural soils^31^, especially during the degradation of plant material^32^, in this study all the plant residues have been removed. Because of these results, we can hypothesize that microbial diversity in soil has a greater impact than OWP amendment or the structure and composition of the soils on even the most highly emitted VOCs. Differences in VOC emissions, however, may also be due to different sample treatment between two studies.

The two most emitted compounds reported in this study are aromatic compounds. Aromatic compounds are released from the shikimate pathway leading to the production of aromatic aminoacids^33,34^. In particular, this pathway is used by microorganisms in order to produce amino acids like phenylalanine, tyrosine, and tryptophan which are used to build proteins.

It has been shown by several studies that microorganisms are a source and a sink of VOCs^35,36^. For instance, *in vitro* soil incubation studies reported that when the concentration of VOCs increased the respiration rate was higher, suggesting that VOCs were used as a nutrient source for some bacteria^20^. In our study we counted more than 250 VOCs having a detectable flux and we found that VOC emissions rates increased with decreasing bacterial diversity.

We further show that VOC emissions were positively correlated with only two prokaryotic phyla (*Proteobacteria* and *Bacteriodetes*), which increased in abundance with the decreasing of the diversity. These data can be interpreted in two non-exclusive ways: (1) either increased VOC emissions are due to the two phyla that are more present than the others, or (2) decreased VOC absorption by the other prokaryotes, which are more abundant. The two interpretations lead to the observed correlation pattern. If the first interpretation is correct, it could be explained by an increased VOCs release by secondary metabolisms sources^37^ which is triggered with the colonization of the space within the microcosms^38^. The second interpretation may be explained by an increased colonization rate of *Proteobacteria* and *Bacteroidetes* which were boosted by the higher concentrations of VOCs in the higher dilution levels where absorbing bacteria take up fewer VOCs^39^.

Studies on biofilters reported that fungi biofilters have a higher efficiency in removing VOCs than bacterial filters^40^. Our results show that fungi that are most present in the most diverse environment are indeed negatively correlated with VOC emissions suggesting they are good VOCs absorbers. These fungi are however not clearly identified in our study as they belong to the “environmental and unclassified” phyla. Other studies have reported that mix of bacteria were however found to be more efficient for absorbing and converting specifics VOCs such as: methanol23 and styrene^41^. Our study tends to demonstrate that bacterial diversity decreases emissions, meaning an increased absorption by most of the bacteria phyla, which is in line with these studies. In view of all these we can hypothesize that a complex microbial structure with a high diversity level might increase the efficiency on converting VOCs also in soil

### Interactions between prokaryotic and fungi diversity

The decrease in density for some fungi phyla and the lower fungi diversity observed at the highest dilution level (D2) might also be the consequence of the competition between fungi and prokaryotes. Probably, fungi have been negatively impacted by the pioneer prokaryotic species. Indeed, prokaryotes secondary metabolisms produced fungistatic VOCs limiting the colonization of the microcosms^42,43^. More specifically, some VOCs were only found in strongly fungistatic soils, and others VOCs had higher concentrations in fungistatic soils^44^. The prokaryotes which emit fungistatic volatiles span a wide phylogenetic spectrum^44^. It has been also reported that several prokaryotes from the *Firmicutes* phylum inhibited the growth of common fungal species^45^. Furthermore, Phenilacetaldheyde, Benzothaziole, Diehtyl sulfide, Dimethyl disulfide and methyl pyrazine, produced by *pseudomonas* sp (*Proteobacteria* phylum), were one of the inhibitors of the fungi mycelia growth and of the spore germination^46,47^. Our results reported a higher relative abundance of *Proteobacteria* in the lowest dilution level (D0) compared to D2 (Fig. 2) and we found that all the fungistatic compounds were emitted around 2 and 10 times more in D2 microcosms than in the D0 (Table 2). Finally, our hypothesis is that the increase of fungistatic VOCs might be related with the increase of the *Proteobacteria* phylum in the highest dilution level (D2).

### Prokaryotic and fungi VOCs emissions profiles

As mentioned above, prokaryotes and fungi are both capable of producing and consuming VOCs. The heatmap (Fig. 7) shows that some phyla are mostly positively correlated with VOC emissions while others are mostly negatively correlated with VOC emissions. A previous study reported that the attribution of a unique VOC emission pattern to a phylum was not possible^48^. For this reason, the aim of this study was not to find common intraspecific VOC patterns. However, it is interesting to underline that data showing increased VOC emission positively correlated with microorganism abundance was observed much less than high VOC emissions associated with low microbial abundance. Other studies found, as in the present study, positive and negative correlations between microorganisms and specific VOCs, and hypothesized two possible VOC-prokaryote interactions for both the positive and negative correlations^49^.

A positive correlation may result from the emission of a given VOC by a given microorganism or from the stimulated growth of this microorganism when that VOC concentration increases. For instance, this correlation could result from the VOCs linked to the quorum sensing like the molecule dimethyl disulfide^38^. Quorum sensing is a system used by microorganisms for intra-specific communication. Quorum sensing microorganisms release specific VOCs as a signal to monitor the cell density^50^. The concentration of these VOCs increases as a function of microorganism growth. In this study, the dimethyl disulfide was positively correlated with the Proteobacteria and Bacteroidetes phyla and was higher when the colonization of those phyla was higher (D2 samples). An example of microbial VOCs promoting the growth of other microorganisms was given by Collimonas pratensis and S. plymuthica (Proteobacteria phylum) species. Those two species induced the growth of P. fluorecens (Proteobcteria phylum) with the collaborative purpose of increasing their chances of survival against other microorganisms in soil^51^.

A negative correlation may result from the absorption of a given VOC by a given microorganism as a carbon source, or from the inhibition of this microorganism when that VOC concentration increases. For the first case it was demonstrated that in carbon-restricted substrate, the growth of Ascomycota phylum was increased from 22% to 45% while exposed to microbial VOCs emission^52^. Whereas an example of inhibition mechanisms was the interaction between Debaryomycetes sp. (Ascomycota phylum) and B. theobromae fungi. When B. theobromae were exposed to the VOCs emitted from Debaryomycetes sp their colonization of the substrate decreased^53^. The same happened to S. pithyophila (Basidiomycota phylum) when exposed to the VOCs emitted from an unidentified mix of bacteria^53^.

**Figure 8:**
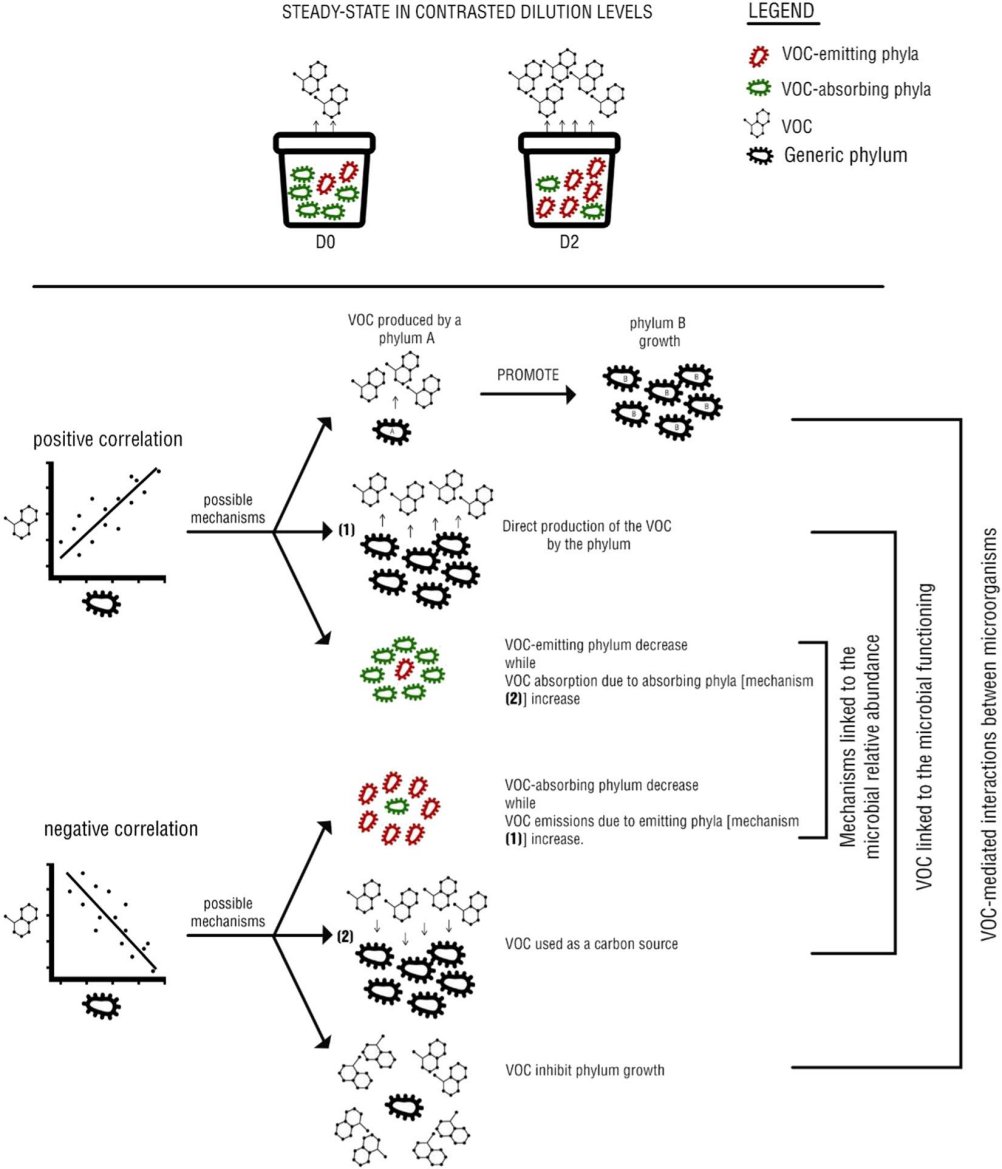
Observation of the steady statement given from the performed analysis (VOCs detection and DNA extraction) of the D0 and D2 samples. The pathways explaining the possible mechanisms behind the positive and the negative correlations are displayed. We hypothesized three different type of pathways (i) Mechanisms linked to the microbial relative abundance: VOCs are not the direct players in the correlation since absorbing phyla decrease while emissions increase due to the increase of the emitting phyla in the microcosms and *vice versa*; (ii) VOC might be linked to the microbial functioning since we hypothesized a direct absorption or production of the VOC by the phylum; (iii) The interaction between microorganisms such as competition and synergy.

These mechanisms can hence imply some VOC-mediated interactions between the microorganisms since the further decrease of VOC concentrations in the soil may be itself linked with the microorganism metabolisms. The positive and the negative correlation between VOC emission and microorganism abundance might also result from a decrease in VOC-absorbing phyla, while VOC-emitting phyla increased and vice versa. For instance, samples D2 reported an increase of the Proteobacteria and Bacteroidetes phyla which were mostly correlated to the VOC emissions. At the same time, the VOC absorbing phyla in D2 samples decrease. Hence, in the D2 samples, Proteobacteria and Bacteroidetes phyla were positively correlated to VOC emission levels, because of the decrease of the absorbing phyla in the microcosms. On the other hand, absorbing phyla were negatively correlated to the VOC emissions, because of the increase of the Proteobacteria and Bacteroidetes phyla. In this case, VOCs are not directly involved in the correlation, but still, be correlated to the phylum. These possible mechanisms are summarized in Fig. 8.

### VOCs mediating interaction between phyla

VOCs were shown to be essential intermediates in the interactions between prokaryotes for nutrients^36^. In particular, some VOCs produced by prokaryotes, that have access to nutrients, are shown to stimulate but suppress the growth of starved bacteria^36^. Compounds tentatively identified in this study as 1,3-butadiene are known to be used as biocontrol agents against other bacteria. In particular, *Pseudomonas* sp. which form part of the *Proteobacteria* phylum can inhibit the growth of *Bacillus* sp, which form part of the *Firmicutes* phylum^52^. In this study, we observed an increase in emission of 1,3-butadiene with dilution levels, while we measured an increase of *Proteobacteria* phylum abundance and a decrease of *Firmicutes* phylum abundance, giving indirect evidence of the effect of 1,3-butadiene in the interaction between these two phyla.

Another interesting compound detected was acetoin (3-hydroxy-2-butanone, m/z 89.056). We detected acetoin in the three dilution levels with an increased relative contribution in the summed VOC with dilution levels. The acetoin is derived from the pyruvate fermentation under anaerobic conditions^53^, and was shown to increase the virulence factors of *Proteobacteria* in the colonization process^54,55^. Other studies have found a positive correlation between the bacteria cell numbers of *Salmonella enterica* (*Proteobacteria* phylum)^55^ with the signal of an unidentified signal from the mass 89, which corresponds to the mass of protonated acetoin. We hypothesize that acetoin could have been a vector promoting *Proteobacteria* growth in reduced microbial diversity conditions. It is interesting to further note that acetoin is a promoter of plant growth inducing systemic resistance in plants^44,53,56^. We can hypothesize that the effect of acetoin on plants may be indirectly linked to promotion of *Proteobacteria* growth.

Our results also showed a doubled emission rate of acetaldehyde and butanone in the most diluted levels compared to the lowest ones. A positive correlation between the number count of *Shigella flexneri* and *Salmonella enterica* (*Proteobacteria* phylum) cells and the emissions of acetaldehyde and butanone have been reported^55^. We can therefore also hypothesize that the increase in acetaldehyde and butanone emissions in D2 might be due to *Proteobacteria* phylum growth in that dilution level.

## Conclusions

This work shows that reduced microbial diversity in soil increased the VOC emissions and decreased the number of VOCs emitted. This work also strongly suggests that there are more bacteria absorbing VOCs than emitting them in soil. Several microbial processes involved in the production of VOCs within the microcosms have been hypothesized, underlining the possibility of primary and secondary metabolism production pathways. Furthermore, our study suggests that *Proteobacteria* and *Bacteroidetes* phyla would be a source of VOCs in soils while other phyla may be mostly sinks. Our results further suggest that positive and negative VOC-mediated interactions between microorganisms may be an important soil colonization process.

## Methods

### Sampling and site description

Samples were collected in the QualiAgro site, a field station taking part of the SOERE-PRO-network (https://www6.inra.fr/qualiagro_eng/Nos-partenaires/The-SOERE-PRO-network). The QualiAgro agronomic set up which is described in this study started in September 1998. The QualiAgro site is located at Feucherolles in northwestern France (35 km west of Paris; 48°52′N, 1°57′E, alt 150 m) on a silt loam textured soil. The soil is classified as a hortic glossic Luvisol (IUSS Working Group WRB, 2014), representative of the Parisian Basin. The main characteristics of these soils are represented by the lack of clay, a silt-loam texture (15.0% clay, 78.3% slit) and an initial pH of 6.9 in the surface horizon (0–30 cm) and good drainage. Moreover, the QualiAgro field experiment is in a cropland dominated region, which leads to low organic carbon and low organic matter concentrations (initial content of 1.1%)^57^.

The experiment was a randomized block design with 4 replicates comparing 4 organic waste products: BIOW (bio-waste compost derived from the co-composting of green wastes and source-separated organic fractions of municipal solid wastes), GWS (compost derived from the co-composting of green wastes with sewage sludge), FYM (farmyard manure) and MSW (municipal solid waste compost derived from the composting of residual solid wastes after removing dry and clean packaging); plus a control without organic input (CN). Samples were collected in 5 blocks of the site amended with mineral N in order to reach optimal N application. Since 1998, the organic waste products (OWPs) have been applied at a rate of ~ 4 t C ha^−1^ every two years on the wheat stubbles in September after harvesting. In each plot, 5 soil cores were randomly sampled at 0–30 cm depth using a core drill and stored in a cold chamber at 4 °C prior to analysis. The sampling was performed in early September 2016, one year after the last amendment of OWPs.

### Microcosms and experimental setup

Soil samples were sieved at 4 mm and homogenized before gamma-ray sterilization (35 kGy; Conservatome, Dagneux, France). The sterility of the irradiated soil was verified by spreading serial dilutions of the soil onto nutrient agar plates. After the sterilization process, soils were inoculated with a diluted soil suspension prepared with the same soil before sterilization^58^. The soil suspension was created by mixing 30 g of soil with 90 mL of sterilized water. From this soil suspension, we used a pure sample (10^0^) D0, and two levels of dilution were prepared with a water ratio of 1:10^3^ (D1) and 1:10^5^ (D2). The second step was the re-inoculation of the sterilized soil with the three different soil suspensions (pure or 10^0^, 10^−3^ and 10^−5^). In order to create the microcosms, 30 g of each sterilized soil sample were transferred to a flask, and we added 50% of the water necessary to reach 60% of the water holding capacity (WHC). We completed the microcosms by adding one of the three soil suspension until 60% of the WHC. Soil samples consisted of soils amended with 4 different OWPs and a control without organic input. Three replicates of each combination of soil and microbial diversity level were prepared, resulting in 45 microcosms in total (n = 45, replicates = 3). Microcosms were sealed hermetically and pre-incubated at 20 °C in the dark. Once a week during six weeks the microcosms have been aerated and the water content was adjusted to maintain constant soil moisture at 60% of the WHC.

One week before the measurement of VOC emissions with the PTR-QiTOF-MS, the silicone flask plugs were substituted with Teflon coated plugs. This was necessary in order to reduce the influence of the emissions of VOCs released from the silicone plugs. Teflon is an inert material reducing VOC emissions that would have been released from the silicone plug. After the six weeks incubation, the microcosms were connected to the PTR-QiTOF-MS in order to measure VOC emissions.

### VOC emissions measurements with the PTR-QiTOF-MS

#### PTR-QiTOF-MS setup

The VOCs were analyzed with a PTR-QiTOF-MS (PTR-Quadrupole Ion guide-TOF, Ionicon, Analytik GmbH, AU). The analyzer setup is described in details by Abis *et al*.^30^ and is only briefly described here. In this study, ionization was carried out with H_3_O^+^ as proton donor. In the drift tube, the pressure was tuned to 4 mbar, the temperature to 80 °C, and the drift voltage to 1000 V. The E/N ratio (Electric field/density of natural particles) was 132 Td where 1 Td is 10^−17^ V cm^2^. The setup of the time of flight timing was: TOF extraction period 40000 ns, pulse width 2000 ns, trigger delay 100 ns. The number of channels was 240.000. This gave a mass spectrum measurement up to 510 m/z. The measurement period was set to 1 s, which means that each sample corresponded to 60 acquisitions of 25000 individual spectra. Raw PTR-ToF-MS data were recorded by TofDaq software (Tofwerk AG, Switzerland).

#### Flask sampling method and flux calculations

Each flask plug was equipped with two PEEK tubes, one allowing the connection with the PTR-QiTOF-MS and the other one was connected with a bottle of dry synthetic air (Alphagaz 1 Air: 80% nitrogen, 20% oxygen, 99.9999%, Air Liquide®). The flasks had a volume of 88 cm^3^. The detection of the VOC emissions from the microcosms was performed during 180 s for every sample. For each sample, an empty flask was used as a reference for zero emissions, using the same Teflon plug as the sample. An air flow (${Q}_{air}$) of 0.3 L min^−1^ (equivalent volumetric flow at 0 °C and 1 atm) of dry synthetic air was passed through a hydrocarbons and humidity filter (Filter for fuel gas, final purity = 99.999%, Restek®) and a Hydrocarbon Trap (Supelco, Supelpure® HC) prior to injection in the flask. A mass flowmeter (Bronkhorst® model F-201CV, accuracy: standard 0.5% Rd plus 0.1% FS, range: 0.2 L min^−1^ to 5 L min^−1^ air) was used to control the synthetic air flow rate. Air was sampled at the chamber outlet into a PTR-QiToF-MS with a 0.05 L min^−1^ flow rate with a 2 m long, 1 mm internal diameter PEEK tube, heated at 80 °C. A measurement cycle consisted in measuring the VOC mixing ratio at the outlet of the flask containing the microcosms (${x}_{VOCmicro}$ in ppb) for 180 s. Then air was sampled for 180 s on the empty chamber with the same Teflon plug sealing to determine ${x}_{VOCempty}$ (ppb). Only the last 60 s of each measurement were kept to calculate averaged mixing ratios in order to ensure a stable VOCs mixing ratio. The VOC emission (${E}_{VOC}$ in nmol g^−1^ s^−1^ dry soil) was calculated as:

1 $${E}_{VOC}=\frac{{Q}_{air}\times ({{\rm{\chi }}}_{VOCmicro}-{{\rm{\chi }}}_{VOCempty})}{{V}_{mol}^{air}\times {m}_{drysoil}}\,$$

where ${V}_{mol}^{air}$ is the air molar volume at standard temperature and pressure (22.4 L mol^−1^ at 0 °C and 1 atm), and ${{\boldsymbol{m}}}_{{\boldsymbol{dry}}{\boldsymbol{soil}}}$ 30 g. After each measurement, the flask was cleaned and the soil transferred in a −40 °C chamber before the DNA extraction.

#### VOCs data analysis

The analysis of the ion peaks in the mass spectra measured with the PTR-QiTOF-MS, the mass calibration, and the processing of the mass table with all the compounds detected were performed using the Spectra Analyser of the PTR viewer 3.1.0.29 software (Ionicon, Analytik GmbH) following the protocol published in Abis *et al*.^30^. Likely isotopes and fragments were identified using a correlation coefficient of 0.99. Therefore, the ions having a time-correlation coefficient higher than 0.99 were considered as either isotopes or fragments depending on the m/z difference. Furthermore, correlated ion peaks that were closer than the resolution of the used PTR-QiTOF-MS were considered a single ion and only counted once.

### Microbial analysis

#### DNA extraction

The DNA extraction has been performed for all microcosms following the protocol developed by GenoSol platform (INRA, Dijon, France, www.dijon.inra.fr/plateforme_genosol) (Terrat *et al*., 2012) for application in large-scale soil survey (Terrat *et al*., n.d.). The protocol consist of mixing in a 15 mL Falcon tube 1 g of each soil sample with 2 g of 100 mm diameter silica beads, 2.5 g of 1.4 mm diameter ceramic beads and 4 glass bead of 4 mm diameter and 5 mL of a solution containing 100 mMTris (pH 8.0), 100 mMEDTA (pH 8.0), 100 mM NaCl, and 2% (wt/vol) sodium dodecyl sulfate. Then, we proceeded with homogenizing the samples in a FastPrep-24 (MP-Biomedicals, Santa Ana, CA, USA) during 90 s and incubated for 30 min at 70 °C before centrifugation at 7000 g for 5 min at 20 °C. The deproteination was performed by collecting 1 mL of the supernatant and incubating for 10 min on ice with 1/10 volume of 3 M potassium acetate (pH 5.5) and centrifuged at 14.000 g during 5 min. The precipitation of the proteins was performed with one volume of ice-cold isopropanol. The last step of the extraction consisted of washing the nucleic acid with 70% ethanol. DNA concentrations of crude extracts were determined by electrophoresis in 1% agarose gel stained with ethidium bromide using a calf thymus DNA standard curve, and used as estimates of microbial biomass (Dequiedt *et al*., 2011). After quantification, nucleic acids were separated from the residual impurities, particularly humic substances, by centrifuging through two types of minicolumn. After quantification, 100 µl of crude DNA extract were separated from the residual impurities, particularly humic substances, by using only the purification steps of Nucleospin Soil kit (Macherey-Nagel GmbH & Co. KG, Düren, Germany). Purified DNA concentrations were finally measured using the Quantifluor (Promega, Lyon, France) staining kit, according to the manufacturer’s instructions.

#### Quantitative PCR (qPCR)

Quantitative real-time PCR was performed on extracted DNA to quantify 16S and 18S rRNA gene sequences^59,60^, which led to the estimation of the F/P ratio. Prokaryotic and fungal quantitative PCR assays were performed using a StepONE (Applied Biosystems, Courtaboeuf, France) with a SYBRGreen® detection system. Purified DNA extract was amplified in a total reaction volume of 20 µl, containing 500 ng of T4 gene 32 protein (MP Biomedicals, France) and 10 µl of Takyon Rox Sybr 2X Mastermix dTTP blue (Eurogentec, Liège, Belgium).

For prokaryotic quantification, the reaction mixtures contained 1 µM of each primer (341 F: 5′ - CCT ACG GGA GGC AGC AG - 3′ and 515 R: 5′ - ATT ACC GCG GCT GCT GGC A - 3′) (López-Gutiérrez *et al*. 2004) and 2 ng of template DNA. The PCR conditions consisted of an initial step of 15 min at 95 °C then 35 cycles of 15 s at 95 °C, 30 s at 60 °C, 30 s at 72 °C and 20 s at 80 °C. The 16S rDNA gene from a pure culture of *Pseudomonas aeruginosa* PAO was used as a standard for the prokaryotic quantitative PCR assay.

Soil fungi were quantified using 1.25 µM of each primer (FR1: 5′-AIC CAT TCA ATC GGT AIT-3′, and FF390: 5′-CGA TAA CGA ACG AGA CCT-3′)^61^, and 2 ng of template DNA. The PCR conditions were: an initial step of 10 min at 95 °C for activation; followed by 35 cycles of 15 s at 95 °C, 30 s at 50 °C and 60 s at 70 °C. Amplified DNA from a pure culture of *Fusarium oxysporum* 47 was used as a fungal standard. These measures were used to estimate the prokaryotic and fungal densities in the samples.

#### High throughput sequencing of 16S and 18S rRNA gene sequences

Prokaryotic diversity was obtained by amplifying a 440-base 16S rRNA from each DNA samples. The corresponding primers were: F479 (5’-CAG CMG CYG CNG TAA NAC-3’) and R888 (5’-CCG YCA ATT CMT TTR AGT-3’) (Tardy *et al*., 2014). The amplification of the DNA was performed during a 25 µL PCR (with 5 ng of DNA for each sample) under the following set up conditions: 94 °C for 2 min, 35 cycles of 30 s at 94 °C, 52 °C for 30 s and 72 °C for 1 min, followed by 7 min at 72 °C.

For fungal diversity, a 350-base 18S rRNA fragment was amplified from each DNA sample (5 ng) with the corresponding primers: FF390 (5’-CGA TAA CGA ACG AGA CCT-3’) and FR1 (5’-ANC CAT TCA ATC GGT ANT-3’)^61^. For each sample, 5 ng of DNA were used for a 25 µL PCR conducted under the following conditions: 94 °C for 3 min, 35 cycles of 30 s at 94 °C, 52 °C for 1 min and 72 °C for 1 min, followed by 5 min at 72 °C.

The purification of the PCR products was performed using the Agencourt® AMPure® XP kit (Beckman Coulter, Italy, Milano) and quantified with the Quantifluor (Promega, Lyon, France) staining kit according to the manufacturer’s instructions. Purified PCR products (7.5 ng of DNA for prokaryotes and 5 ng of DNA for fungi) were amplified twice in order to integrate to the 5′ end of the primers a 10-bp multiplex identifiers allowing the specific identification of the samples and the prevention of PCR biases. For prokaryotes, the second PCR conditions were the same as previously described but with only seven cycles. For fungi, the second PCR conditions were optimized, with the number of cycles being reduced to seven and the denaturation step processed at 94 °C during 1 min. PCR products were purified with the MinElute PCR purification kit (Qiagen NV) and quantified with the Quantifluor (Promega, Lyon, France) staining kit according to the manufacturer’s instructions. Equal amounts of each sample were pooled and then cleaned with the SPRI (Solid Phase Reverse Immobilization Method) using the Agencourt® AMPure® XP kit (Beckman Coulter, Italy, Milano). The pool was finally sequenced with a MiSeq Illumina instrument (Illumina Inc, San Diego, CA) operating with V3 chemistry and producing 250 bp paired-reads.

#### Bioinformatic analysis of 16S and 18S rRNA gene sequences

The bioinformatics analyses were performed using the GnS-PIPE developed by the Genosol platform (INRA, Dijon, France) (Terrat *et al*., 2012). At first, all the 16S and 18S raw reads were organized according to the multiplex identifier sequences. All raw sequences were checked and discarded if: (i) they contained any ambiguous base (Ns), (ii) if their length was less than 350 nucleotides for 16S reads or 300 nucleotides for 18S reads, (iii) if the exact primer sequences were not found (for the distal primer, the sequence can be shorter than the complete primer sequence, but without ambiguities). A PERL program was then applied for rigorous dereplication (i.e. clustering of strictly identical sequences). The dereplicated reads were then aligned using Infernal alignment^62^, and clustered into operational taxonomic units (OTU) using a PERL program that groups rare reads to abundant ones, and does not count differences in homopolymer lengths with a threshold of 95% of sequence similarity. A filtering step was then carried out to check all single-singletons (reads detected only once and not clustered, which might be artifacts, such as PCR chimeras) based on the quality of their taxonomic assignments. Finally, in order to compare the datasets efficiently and avoid biased community comparisons, the reads retained were homogenized by random selection (23 700 and 11900 reads for 16S and 18S rRNA gene sequences, respectively). The retained high-quality reads were used for: (*i*) taxonomy-independent analyses, determining the Shannon index (*ii*) taxonomy-based analysis using similarity approaches against dedicated reference databases from SILVA^63^. The raw datasets are available in the EBI database system under project accession number PRJEB29286.

### Statistical analysis

Microbial biomass, microbial diversity Index (Shannon) and the relative abundance of prokaryotes, and fungal *phyla* in the microbial composition were processed by the ANOVA test. With the ANOVA test, we also analyzed the effect of the organic waste product on the microbial community for the different dilution level The dataset before the statistical analysis was made of 754 variables (number of peaks detected) and 45 samples comprising the 3 replicate for each sample. In order to select the most representing variables of the dataset, several statistical tests have been performed using the R software (Version 1.0.153 – © 2009–2017 RStudio). At first, the normality test (Shapiro-Wilk test, W > 0.9) has been applied to verify that the mean mixing ratios were normally distributed for each VOC. Next, the homogeneity of the variances was verified for each treatment using the Levene test. Once the normality and the homogeneity of the variances were validated, a t-test was performed in order to see if the VOC flux was significantly higher than 0. Correlated masses that were closer than the resolution of the used PTR-QiTOF-MS were removed in order to not count twice the same peak. Finally, an ANOVA test was performed. The final selected dataset comprised of 45 samples and 239 variables meaning that only 32% of the dataset was kept for further analysis. The ANOVA tests were followed by the Tukey post hoc test.

A principal component analysis (PCA, Package *Ade4* Version 1.0.153 – © 2009–2017 R Studio) was performed to see the different VOCs compounds differentiating the three dilution levels. The Shannon index of diversity (Figs. 1 and S4) was calculated with the diversity function of the vegan package (version 2.4-3) in the R software (version 3.2.3). The diversity index was calculated as $H\,=\,\sum _{VOC}{E}_{VOC}\,\log ({E}_{VOC})$, where the sum is over all VOCs recorded in the mass table. The correlation matrix between VOCs and microorganisms has been created selecting the VOC having a R^2^ of the correlation microorganisms/VOC larger than 0.6, the final number of compounds displayed was 107.

Microbial biomass, microbial diversity Index (Shannon) and the relative abundance of prokaryotic, and fungal *phyla* in the microbial composition were processed by the ANOVA test. With the ANOVA test, we also analyzed the effect of the organic waste product on the microbial community for the different dilution levels. All significant effects were assessed by Tukey’s Honestly Significant Difference (HSD) *post hoc* test (*P* < 0.05).

The correlation between the summed VOC emissions and VOC diversity and the different microbiological factors has been calculated using the basic package STAT of R studio. We choose the Spearman correlation as a method since the distribution of the dataset was not normal.

To visualize the relationship between VOCs and soil physico-chemical and microbiological factors, redundancy analysis (RDA) and variance partitioning were carried out. All statistical analyses were performed with the Vegan package (v.2.0-8)^64^ in R software version 3.0.1. RDA was applied to VOCs variables (diversity and total emission rates) using soil physico-chemical (Corg, pH, C/N) and microbial community parameters (molecular microbial biomass, prokaryotic and fungal richness, prokaryotic and fungal diversity, F/P ratio) as explanatory variables. At first, a global variance partitioning was applied to quantify the respective effect of soil physico-chemistry and soil biology (rda followed by anova.cca function). Then, a detailed variance partitioning provided the pure effect of each exploratory variable was proceeded (ordiR2step followed by anova function)^65^. The interactions between all explanatory variables were also included in the model. The significance of the model and of each explanatory variable included in the model was tested using 10000 permutations.

## Supplementary information

Supplementary material.

## Data Availability

Data are available in data.inra.fr website (10.15454/4W9DM2).
