# Supplementary material for: Reduced microbial diversity induces larger volatile organic compound emissions from soils
Source: Sci Rep. 2020 Apr 8;10:6104. doi: 10.1038/s41598-020-63091-8 (PMC7142124; doi:10.1038/s41598-020-63091-8)
Supplement: Supplementary file 1 — Supplementary material. [file 41598_2020_63091_MOESM1_ESM.docx]

**Reduced microbial diversity induces larger volatile organic compound emissions from soils.**

Letizia Abis ^1,2 *^, Benjamin Loubet^2^, Raluca Ciuraru^2^, Florence Lafouge ^2^, Sabine Houot^2^, Virginie Nowak^3^, Julie Tripied^3^, Samuel Dequiedt^3^, Pierre Alain Maron^3^, Sophie Sadet-Bourgeteau ^3^

^1^ Sorbonne Université, UPMC

^2^ INRA, UMR ECOSYS, INRA, AgroParisTech, Université Paris-Saclay, 78850, Thiverval-Grignon, France

^3^INRA, UMR AgroEcologie, AgroSup Dijon, BP 87999, 21079 Dijon cedex, France

^*^ Now working at: Technische Universität Berlin, Umweltchemie und Luftrinhaltunz, Straße des 17. Juni 135, Berlin, 10623, Germany.

Corresponding author: letizia.abis@tu-berlin.de


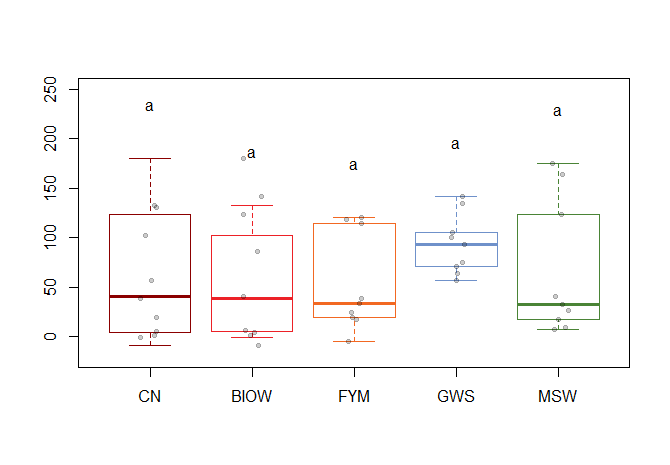


nmol/(s g(sol sec) 10^-4^)

**Figure S1**. Summed VOCs emission rates per soil treatment. BIOW= bio-waste compost, MSW= municipal solid waste compost, CN= control without organic input, FYM= farmyard manure, GWS= green waste and sludge compost, D0= microbial diversity pure or 10^0^, D1= microbial dilution diversity equal to 10^-3^, D2= microbial dilution diversity equal to 10^-5^.

**(a)**


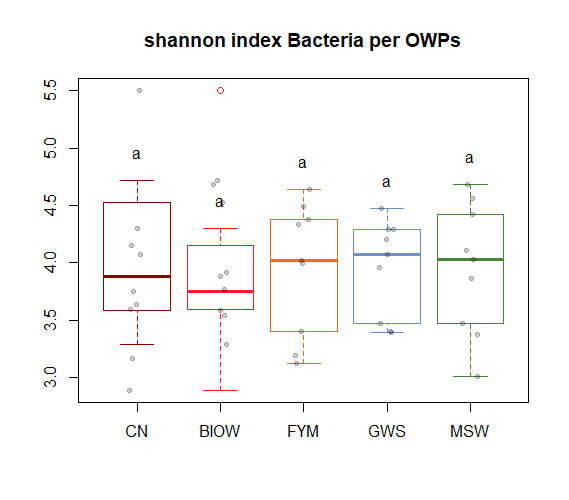


Shannon Index

**(b)**


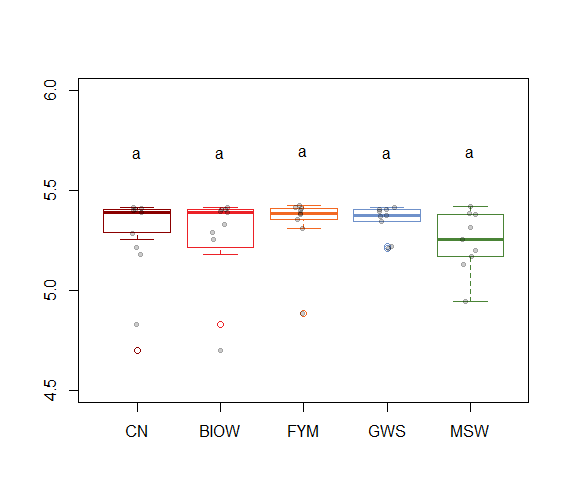


Shannon Index

**Figure S2**. (a) Shannon index for prokaryotes in soil per OWPs. Bold line=median, boxes= interquartile, whiskers = minimum and maximum. Point= value the Shannon Index for each sample. Letters indicate significant differences according to the Tukey test with p.value >0.05. (b) Shannon index for VOCs emissions in soil per OWPs. D0= microbial diversity pure or 10^0^, D1= microbial dilution diversity equal to 10^-3^, D2= microbial dilution diversity equal to 10^-5^.


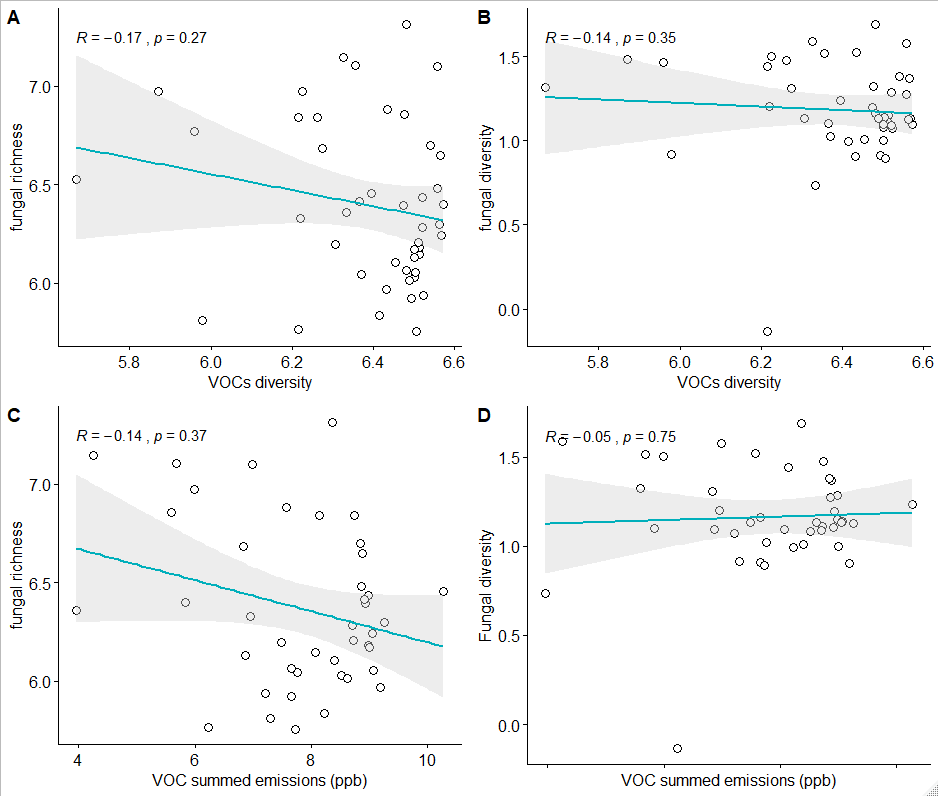


**Figure S3.** Correlation between log (VOC diversity) and a) log (fungal richness), b) log (fungal diversity) an log (VOC summed emissions) and c) log (fungal richness), d) fungal diversity. The Spearman correlation coefficient (R) and the p.value (*p*) are also displayed for all correlations.
